# Resection of Eloquent Located Brain Tumors by Mapping Only—A Feasibility Study

**DOI:** 10.3390/brainsci13101366

**Published:** 2023-09-25

**Authors:** Franziska Staub-Bartelt, Marion Rapp, Michael Sabel

**Affiliations:** Department of Neurosurgery, University Hospital Duesseldorf, 40225 Düsseldorf, Germany

**Keywords:** brain mapping, monopolar stimulation, bipolar stimulation, eloquent brain tumor surgery

## Abstract

Background: Patients with eloquently located cerebral lesions require surgery that usually employs mapping and monitoring techniques for the preservation of motor and language function. However, in many cases, mapping only might be sufficient, reducing the need for technical and personnel logistics. Here, we report our experiences using a device that can be operated by the surgeon independently, providing mapping techniques but omitting monitoring techniques. Methods: For monopolar and bipolar cortical/subcortical stimulation, pre-set programs were available and intraoperatively used—two enabling EMG real-time tracking of eight muscles for monopolar (cortical/subcortical) mapping, and two programs for 60 Hz stimulation, one with EMG and one without. Motor mapping was performed under continuous observation of the screened EMG signal and acoustic feedback by the surgeon. For the 60 Hz stimulation, a standard bipolar stimulation probe was connected through a second port. The preoperative application of the subdermal EMG needles, as well as the intraoperative handling of the device, were performed by the surgeons independently. Postoperatively, an evaluation of the autonomous handling and feasibility of the device for the chosen test parameters was conducted. Results: From 04/19–09/21, 136 procedures in patients with eloquently located cerebral lesions were performed by using the “mapping-only” device. Mapping was performed in 82% of the monopolar cases and in 42% of the bipolar cases. Regarding the setup and sufficiency for the cortical/subcortical mapping, the device was evaluated as independently usable for motor and language mapping in 129 procedures (95%). Gross total resection was achieved, or functional limit throughout resection was reached, in 79% of the patients. 13 patients postoperatively suffered from a new neurological deficit. At the 3–6-month follow-up, three patients showed persistent deficit (2%). All of them had language disturbances. The setup time for the device was less than 7 min. Conclusions: The device was evaluated as sufficient in over 90% of cases concerning monopolar and bipolar mapping, and the setup and handling was sufficient in all patients. With the present data we show that in well-selected cases, a very simple system providing mapping only is sufficient to achieve gross total resection with the preservation of functionality.

## 1. Introduction

Maximum resection is crucial for favorable outcomes in patients with intrinsic brain tumors [1,2,3], but is also found to be important in patients with other histopathological entities [4,5,6,7]. In the past decades, numerous technical innovations have been introduced to facilitate the function-preserving resection of different brain tumor entities. Intraoperative neuromonitoring (IONM-o) and mapping (IONM-a) procedures of the cortex and subcortical structures, for example, have been complemented by fluorescence-guided procedures [8,9] and intraoperative MRI techniques [9]. Also, preoperative planning with mapping techniques via transcranial mapping [10,11,12] and diffusion weight imaging [13,14] has become more complex in order to increase safe maximum resection.

Particularly, intraoperative techniques like IONM-o and IONM-a by direct cortical and subcortical mapping techniques have been established widely in neurooncological surgery for the resection of tumors relating to cortical and subcortical functional areas [12,15,16,17,18], as they provide real-time monitoring of the patient’s motor and language functional integrity. IONM-o is applied for the monitoring of motor and sensory function. Motor evoked potentials (MEP) are employed for the monitoring of motor integrity. These evoked muscle responses are captured and analyzed, providing insights into the functional status of the motor pathways in patients who are asleep [19,20,21,22]. Sensory pathway integrity is monitored by somatosensory evoked potentials (SSEP), measuring the latency and amplitude of two signals between defined points. The underlying principle of SSEP monitoring involves the generation and recording of electrical signals that reflect the transmission of sensory information along neural pathways [15,23]. Thus, these technically and logistically demanding techniques play a crucial role in complex brain surgery where the risk of functional tissue damage is high, and where avoiding such damage while achieving the maximum extent of resection is the main goal in brain tumor surgery [24,25,26].

Besides these monitoring techniques, there is the possibility of intraoperative brain mapping. This can be done with a high-frequency stimulation technique using a monopolar stimulation probe as well as a low-frequency stimulation technique using a bipolar stimulation probe. Today, monopolar stimulation is the standard for monitoring motor functionality [27,28]. Language mapping is standardly performed by bipolar low-frequency stimulation due to its longer stimulus duration.

For IONM-o with MEP and SSEP monitoring, specially trained personnel are needed to operate technical devices, evaluate the collected measured values, and pass them on to the surgeons. These data serve the surgeon as the basis for further decisions regarding the surgical procedure. The use of bipolar stimulation requires a simple stimulation device (e.g., Ojeman stimulator) and a person who coordinates and evaluates the language test performance. Monopolar stimulation requires the monopolar stimulation device, a reference electrode, and the EMG recording. This technique is usually provided by IONM-o machines, which also provide SSEP, MEP, and ECOG options.

Due to the introduction of a small new device which allows both monopolar and bipolar mapping by the surgeon, a pilot project was started in our clinic. In this study, we report on the indications of “mapping only” without monitoring devices, the neurological outcomes, and the postoperative evaluation of whether the “mapping only” strategy that was determined preoperatively was also confirmed intraoperatively as sufficiently safe by the surgeons.

## 2. Patients and Methods

The present analysis was conducted between January 2019 and January 2023 at the Department of Neurosurgery at University Hospital Düsseldorf, with approval from the local ethical committee (Study Number 2022–2242). The study focused on evaluating intraoperatively collected data from cortical and subcortical mapping procedures performed on patients with eloquently located supratentorial brain lesions. We also conducted analyses on neurological outcomes, postoperative MRIs, and epidemiological data.

During all surgeries, we utilized the C2 Xtend, and later, the C2 Xplore devices (inomed Medizintechnik GmbH in Emending, Germany). These devices, particularly the C2 Xplore, were a novel addition at the beginning of this study, offering a wide range of functions, concerning brain mapping procedures. We have enclosed a figure illustrating the C2 Xtend device (Figure S1A) and an intraoperative screenshot under monopolar stimulation (Figure S1B). Consequently, the primary objective of our analysis was to determine whether these advanced devices could effectively replace the standard equipment typically used for mapping and monitoring procedures in cases involving eloquently located brain lesions.

### 2.1. Patients

Inclusion criteria for the present analysis were as follows: (1) supratentorial brain tumor surgery in patients >18 years between January 2019 and January 2023, and (2) the use of the C2 Xtend or C2 Xplore device during the surgery.

All patients that underwent surgery using the mapping-only device in the reported period were included, notwithstanding the assumed neuropathological diagnosis and surgery mode (resection vs. open biopsy). If an open rather than stereotactical biopsy was planned for lesions involving assumed high eloquent localizations, the mapping device was used for the definition of the biopsy limits. This is particularly important in patients with assumed high-grade intrinsic tumors, as the extent of resection is directly linked to the outcome of patients. If the preoperative MRI screening revealed a clear vascular conflict of the tumor, e.g., a tumor involving the Sylvian fissure, patients underwent the surgical procedure using the established IONM-o processes, including MEP and SSEP monitoring, and therefore were not included in the analysis.

### 2.2. Methods

#### 2.2.1. Mapping Data

In the present cohort, two devices were used for intraoperative brain mapping procedures: firstly, the C2 Xtend, and later, the successor model C2 Xplore (inomed Medizintechnik GmbH, Emmending, Germany, Neuro Explorer Software Version 6). Both devices enable cortical and subcortical brain mapping. The setup and intraoperative handling of the device is conducted by the surgeon alone without the need of additional external staff.

#### 2.2.2. Setup and Implementation

We implemented a standardized configuration and provided training to all neuro-oncology surgeons to ensure consistency in our procedures. This technical setup involved the preoperative placement of subdermal needles for an 8-channel electromyography (EMG) system. Individual customization is possible for the muscles to be monitored. At our clinic, during intraoperative monitoring via EMG, we focused on assessing the muscles located on the contralateral side of the lesion, which are listed below.

#### 2.2.3. Face: *M. orbicularis* Oris, *M. mentalis*

Upper extremity: *M. biceps brachii*, *M. abductor pollicis breves*, and hypothenar muscle group.

Lower extremity: *M. quadriceps femurs*, *M. tibialis anterior*, and *M. abductor hallucis*.

In addition, a neutral electrode was placed in the deltoid muscle and a reference electrode was placed in the FC position according to the 10–20 system. If there was a conflict with the chosen skin incision, the reference electrode was positioned accordingly. After insertion of the needles, they were connected to an adapter box.

All presurgical preparations, including the EMG setup, were performed by the operating surgeon. The setup time for the device and needles was measured randomly.

#### 2.2.4. Stimulation Settings

We stored various programs with the corresponding standard settings on the device. These can also be individually set and configured. At our department, we chose the same parameters as used in our IONM-o systems.

The monopolar cortical stimulation was performed either cortically or subcortically with a monopolar probe. In both operational modes, we employed continuous stimulation utilizing a repetition rate of 0.5 ms. Stimulation was administered in the form of a stimulus train, consisting of five individual pulses, commonly referred to as a “train of five”. The interval between successive pulses within this train was set at 4 ms, while each individual pulse had a width of 500 µs. During the monopolar stimulation, the EMG was continuously transmitted to a large display for the visual control of the triggered muscles; in addition, acoustic feedback was triggered via an EMG signal. There were two different programs available: one for cortical and one for subcortical stimulation.

Bipolar stimulation was performed either cortically or subcortically as well. For stimulation, a range of 0.5 to 4 mA with a pulse width of 0.8 mA was used. Each stimulation cycle consisted of a single pulse, delivered at a frequency of 60 Hertz. The stimulation duration was set at 4 s for each cycle.

During the resection and stimulation, language testing was performed using a standardized protocol with various types of test tools, covering different aspects of language. A more detailed description of this very specific test battery is beyond the scope of this paper.

For the monopolar mapping, the stimulation of the motor cortex standardly began at 10 mA (with the upper limit capped at 20 mA). Once the cortical thresholds for positive responses in the EMG were determined, we proceeded to test the cortical region encompassing the underlying lesion. If there was positive testing in the area of the surgical approach, a new threshold for this specific area was established. Depending on these results, the corticotomy and subcortical preparation could be initiated. The subcortical testing phase takes place during the resection procedure.

The specific program is chosen pre-surgically by the surgeon at the monitor. Programs could be changed throughout surgery.

Parameters related to the stimulation intensity, repetition rates, the quantity of stimuli, interpulse stimulation intervals, and pulse widths were all adjustable to accommodate the preferences and experience of the user. These adjustments could be made at any point during the procedure according to specific requirements.

If the device was under a sterile drape, it could be operated by the surgeon only during the surgery.

### 2.3. Awake Surgery

Awake surgery is a standard procedure in our department for patients with tumors located in the left frontal or temporal lobe, in order to be able to test for language disturbances during resection. We also indicated awake surgery for patients that had to undergo fine-motor skill testing during surgery or for vision control.

### 2.4. Evaluation of Sufficiency

Surgeons were interviewed directly in the postoperative phase if the applied technique was sufficient in their view. Comments were collected and grouped into an evaluation of “sufficient” or “non-sufficient” procedures.

### 2.5. Evaluation of Neurological Outcome

Upon admission, patients underwent an initial neurological examination in order to maintain comparability. Following surgery, patients were subject to multiple evaluations, particularly if any new neurological deficits arose. For the current analysis, we consistently utilized the examination conducted at the point of discharge to define the postoperative assessment. Furthermore, patients who developed new neurological deficits in the postoperative period were subsequently monitored at approximately 3 months and 6 months following surgery. Permanent deficit was defined by a persistent deficit at the 6-month follow-up.

### 2.6. Residual Volume (MRI)

To evaluate the remaining tumor volume, we conducted a review of the postoperative MRI scans. All MRIs were conducted within a 72-h window following surgery. Our classification system included four distinct groups for describing the results: (1) the macroscopic total resection and total resection in the postoperative MRI, (2) the macroscopic total resection and residual tumor volume in the postoperative MRI, (3) the macroscopic residual tumor volume and residual tumor volume in the postoperative MRI, and (4) no MRI.

The residual volume was calculated by one member of the study team by usage of a volumetry tool within the local radiology information system (SECTRA Workstation 101, IDS7, Version 24.1, Sectra AB, Sweden, 2022). The results of the residual tumor volume were expressed in mL, with volumes less than 0.1 mL defined as indicative of gross total resection.

Sociodemographic information, along with the neuropathological diagnosis and any pertinent medical/surgical histories, were extracted from the local patient administration system. Neuropathological findings predating the introduction of the WHO 5 Classifications of Central Nervous System Tumors in 2021 [29] were modified to conform to the revised classification criteria.

### 2.7. Statistical Analysis

A statistical analysis was performed using IBM SPSS Statistics Version 26 (IBM Corporation, Armonk, NY, USA).

Data were tested using the Shapiro–Wilk Test for normal distribution. Cohort data were not normally distributed; therefore, non-parametric testing with Pearson’s chi-squared test for nominal variables, and the independent samples *t*-test, were performed. Statistical significance stated as a *p*-value for all results was set at 0.05.

## 3. Results

During the specified screening period from January 2019 to January 2023, the mapping devices were employed in 136 procedures. These procedures were carried out on 131 patients, with a mean age of 56 with a standard deviation of ±16 years at the time of their initial recorded surgery. The age range spanned from 22 to 86 years. Of the patients, 62 (47%) were female, while 69 (53%) were male.

Predominant neuropathological diagnoses were metastasis (35%, n = 34), with an equal distribution of Glioblastoma WHO grade 4. In terms of lesion location, 75 procedures were performed on lesions within the left hemisphere (55%), while 59 procedures were directed at right hemisphere tumors (43%). Moreover, two procedures involved surgery at the splenium (2%). Unexpectedly, intraoperative vascular conflicts were encountered in two patients. An overview of the cohort is presented in Table 1.

**Figure 1:**
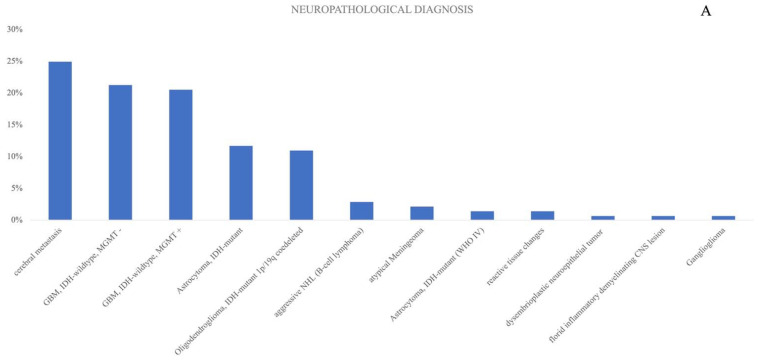

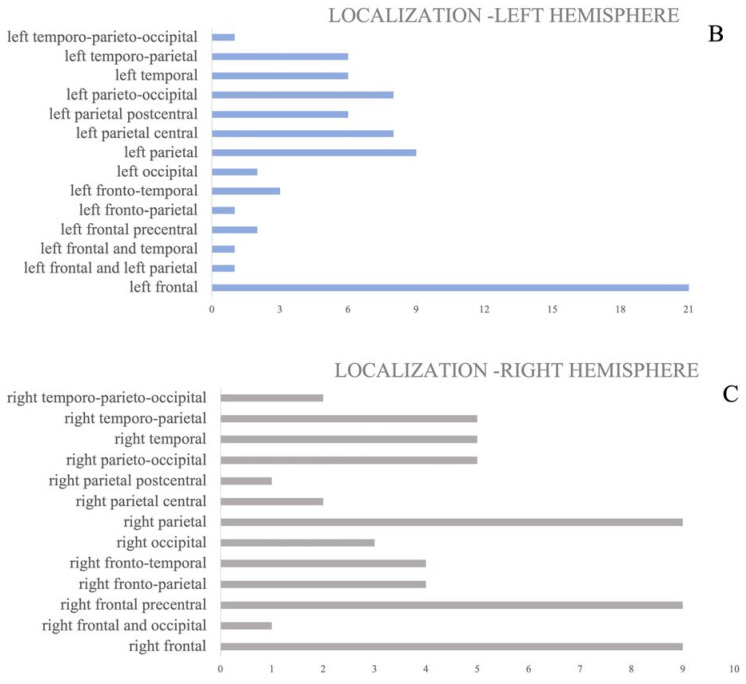

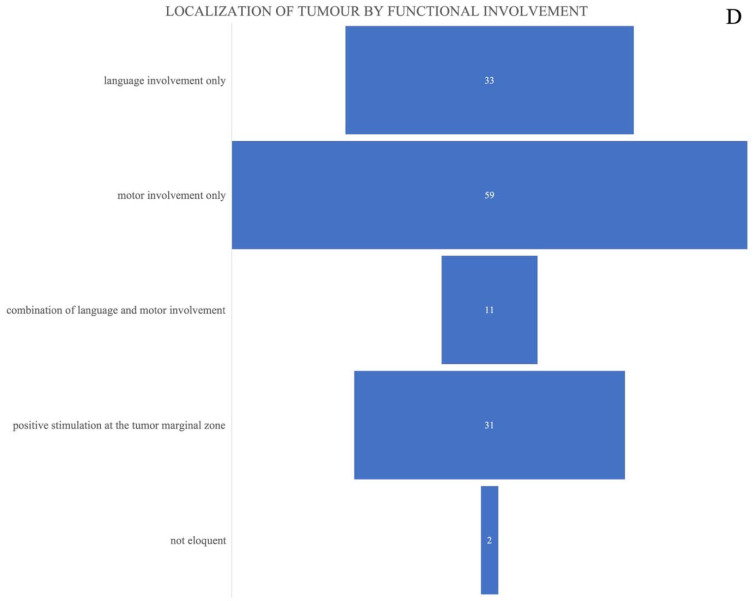
(**A**) percentage distribution of diagnosis within the cohort, (**B**) visualization of detailed tumor location in the left hemisphere, (**C**) details for tumor localization for right hemispheric tumors. (**D**) Details of tumor localization concerning the functional involvement.

Figure 1A–D visualizes different neuropathological details and details about the localization of the lesions that were included in the study, as well as the functional involvement of the tumors that were surgically treated. Tumor localization was grouped into four functional involvements: “language involvement only”, “motoric involvement only”, “combination of language and motor involvement”, as well as “positive mapping only at the tumor margin”. The last group defined tumors, that might only have been partly functionally located, as we only received positive mapping/stimulation results at the tumor margins, sometimes with high current thresholds.

53% (n = 72) of all procedures in the cohort were performed as awake procedures, independent from localization or mapping techniques. Left-hemispheric lesions more often triggered awake status, with 56 procedures conducted in the awake setting in lesions located in the left-hemisphere, versus 16 procedures in right hemisphere lesions (78% vs. 22%; *p* < 0.01). All procedures (n = 2) with lesions located at the splenium were performed as awake surgeries. Patients with tumor-vascular conflicts in the preoperative MRI were excluded, adhering to the defined exclusion criteria. Nevertheless, in two patients, there was an unexpected vascular conflict due to a tumor extension to the vascular level of the Sylvian fissure, which was not seen in the preoperative MRI scan. The mean setup time (n = 48) was 5 min 48 s.

### 3.1. Mapping Data of Monopolar Stimulation

In 111 procedures (82%), monopolar mapping was conducted by the surgeons. 24 procedures (18%) were performed without planned monopolar mapping. In one surgical case, there was partly missing documentation regarding the use of the monopolar mapping technique (information on cortical stimulation was present but information on subcortical stimulation was missing). Cortical monopolar mapping was performed in 111 procedures (82%), of which 87% (97) could define the cortical motor threshold. In the seven remaining cases, the cortical monopolar stimulation up to 20 mA remained negative regarding the EMG responses.

**Figure 2:**
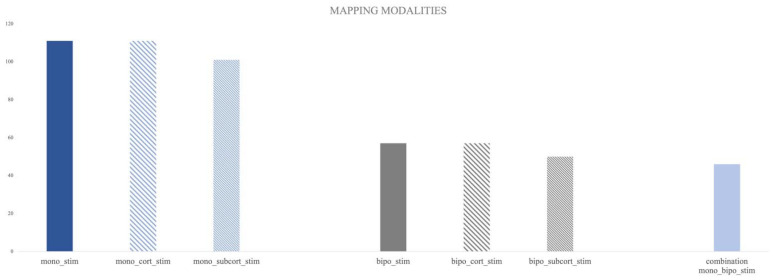
Overview of mapping modalities used in the cohort: mono_stim = monopolar stimulation, mono_cort_stim = monopolar cortical stimulation, mono_subcort_stim = monopolar subcortical stimulation, same abbreviations were used for bipolar stimulation).

Subcortical monopolar stimulation was carried out in 101 surgical cases (74%). In 10 procedures, no tracking of the corticospinal tract defined as a positive EMG response was achieved, with a stimulation intensity of up to 15 mA (Figure 2).

The mean cortical stimulation intensity for the cohort was 7.7 mA [±4.3 SD], with a range of 1.5–20 mA, and the subcortical stimulation intensity was 4.4 mA [±3.6 SD] ranging from 0.3–15 mA.

### 3.2. Mapping Data of Bipolar Stimulation

Overall, 57 procedures were conducted with bipolar stimulation (42%). Bipolar cortical stimulation was employed in 42% of the cases (n = 57); however, in eight cases, there was no stimulation-induced effect on the control area (14%). Furthermore, 29% of the procedures used subcortical bipolar stimulation (n= 50), of which, in 15 cases, only negative stimulation results (no speech arrest or other disturbances in speech testing) were achieved (Figure 2).

The mean cortical bipolar stimulation intensity was 1.3 mA [±0.6 SD], ranging from 0.5 to 3 mA, and the subcortical bipolar stimulation intensity was 1.2 mA [±0.4 SD], with an observed range from 0.8 to 2 mA. At this point, it is important to notice that, due to technical differences in comparison to standard bipolar stimulation devices, including the Ojeman stimulator and monitoring devices with separate mapping boxes that measure the peak current, the device used measured the peak-to-peak current, leading to a displayed output stimulation intensity twice as high as those of standard devices (e.g., if the peak current is displayed as 0.5 mA, the peak-to-peak current is displayed with 1 mA). For the present analyses, the stimulation intensity results were recalculated as the peak current for better comparability to other IONM-o/IONM-a devices.

Bipolar stimulation was used significantly more often in left-hemisphere tumors than right-hemisphere lesions (*p* = 0.011).

A combination of both mapping modalities was used in 46 (34%) surgical procedures.

In regard to the preoperatively defined required mapping or monitoring techniques, in 95% (n = 129) of the procedures the choice of the “mapping-only” procedure was evaluated as sufficient by the performing surgeon. In this sub-cohort, in three cases additional monitoring via strip electrode was evaluated as “would have been helpful” but did neither influence the surgical outcome regarding the extent of resection nor the neurological outcome of the patients. In five cases, the “mapping-only” procedure was evaluated as “not sufficient”. In two of the cases, there were technical issues leading to technically no stimulation. In three cases, MEP/SSEP monitoring was intraoperatively evaluated as obligatory. In two of those cases, a marginal residual tumor volume (0.1 mL) was revealed in the postoperative MRI. In two further cases, all applied mapping techniques remained negative; thus, the tumour localization appeared as not functional during surgery and mapping was evaluated as “not needed”. An evaluation of the sufficiency of the “mapping-only” procedure did not significantly depend on localization (*p* = 0.255), but in cases when awake surgery was planned but patients were not adequately awake, the “mapping-only” procedure was significantly more often evaluated as “insufficient” (*p* = 0.042).

### 3.3. Neurological Outcome

#### 3.3.1. Postoperative Neurological State

**Figure 3:**
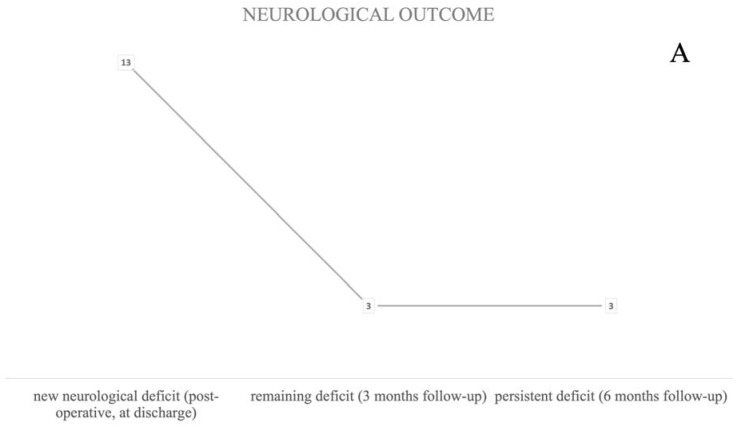

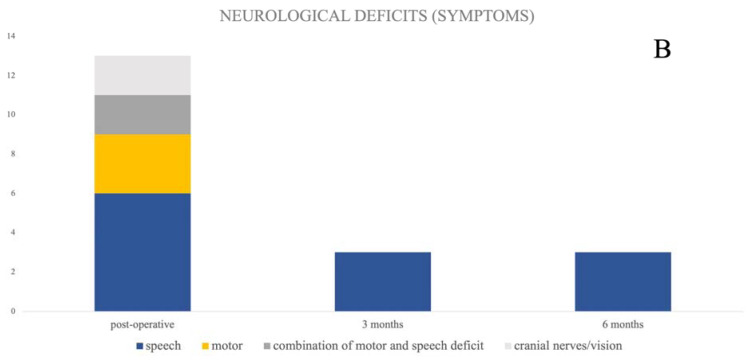
Number of new neurological deficits related to surgical intervention at three time points (“postoperative”, “three-month”, and “six-month follow-up”) (**A**) and their corresponding symptoms at the corresponding time point (**B**).

Neurological deterioration, defined as a new neurological deficit in the postoperative phase, was seen in 10% of the procedures (n = 13). Two patients died in the postoperative course; however, death was not directly associated to surgical intervention (Figure 3A). Localization did not significantly influence a direct postoperative deficit, however a trend for left-hemispheric lesions was seen (*p* = 0.099), whereas bipolar stimulation triggered new postoperative deficits more often (*p* = 0.008).

The majority of patients suffered from new speech disturbances (46%, n = 6). Motoric impairment was seen in three patients (23%). A combination of new motor and speech deficits was seen in two patients (Figure 3B).

#### 3.3.2. 3 Month FU

After three months, three (2.2%) patients still suffered from a neurological deficit, one more patient had died, and two patients did not show up to follow-up appointments and were therefore categorized as loss of follow-up (Figure 3A). A persistent, respectively permanent deficit at the 6-month follow-up was still recorded in those three patients (2.2%). All of them suffered from a persistent speech disturbance. There was no permanent motor deficit in this cohort (Figure 3A,B) under mapping-only conditions.

#### 3.3.3. Permanent Deficit

Permanent deficits were not caused by any vascular complications such as infarction or bleeding. In all three patients with permanent deficit, a combination of monopolar and bipolar stimulation was used. There was a trend for a higher risk of postoperative deficits in patients who underwent awake surgery and in cases where awake surgery was planned but the patients did not wake up adequately (*p* = 0.53).

### 3.4. Resection Results as Evaluated by Postoperative MRI

In 127 procedures, a postoperative MRI was obtained (93%). Nine procedures were conducted as open biopsies and an MRI scan was not planned and therefore also not conducted in the postoperative course.

**Figure 4:**
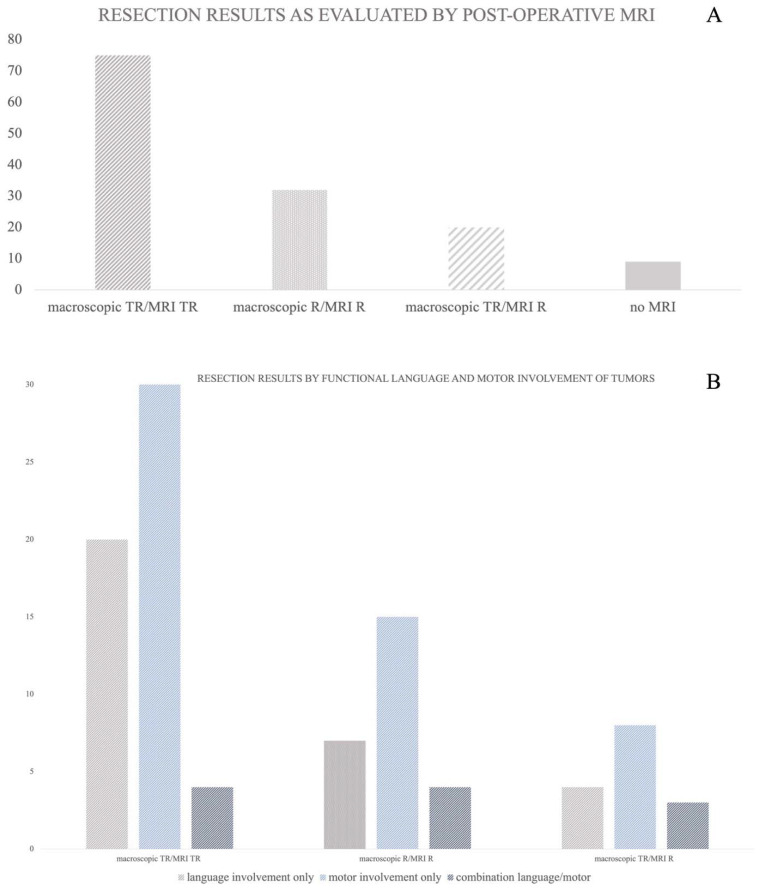
(**A**,**B**) A: Resection results evaluated by 72 h postoperative MRI scans. 127 MRIs were available for this analysis. In the majority of patients either gross total resection or expected residual volume was confirmed (79% macroscopic TR/MRI TR and macroscopic R/MRI R). In 20 procedures an unexpected residual volume was revealed with a mean volume of 0.47 mL (macroscopic TR/MRI R).

Concerning the whole cohort, 59% (n = 75) of the postoperative MRI scans showed no contrast enhancement (threshold defined as <0.1 mL), and an intraoperative evaluation of the gross total resection was confirmed. However, in 25% (n = 32) of all procedures, there was an expected residual tumour volume, as throughout the mapping process functional limits were defined and the resection had to be stopped at some point. In the remaining 16% (n = 20) of procedures, postoperative MRI scans revealed an unexpected residual volume with a mean residual volume of 0.47 mL [±0.7 SD] ranging from 0.1 to 2.8 mL. The mapping modality did not significantly correlate with the resection result (monopolar stimulation *p* = 0.303, bipolar stimulation *p* = 0.309). Additionally, there was no significant correlation between the resection result and the sufficiency of the mapping procedure (*p* = 0.114). Figure 4A,B illustrates resection results of the cohort, as well as the resection results divided into groups of functional involvement. A total of 51% of the tumors involving motor pathways showed a postoperative total resection (as defined by contrast enhancement <0.1 mL in the postoperative MRI), as did 61% of the tumors involving language, and 36% of the tumors combining motor and language functionality. Surgery was stopped according to functional restrictions obtained by the mapping procedure due to motor impairment in 25% of the procedures, language affection in 21% of the procedures, and a combination of both motor impairment and language affection in 36% of the procedures (Figure 4B). Figure 4B illustrates the resection results grouped by the functional involvement of tumors.

## 4. Discussion

In the present study, mapping-only surgical approaches that were performed with a device independently handled by the surgeon, are described for the resection of eloquently located supratentorial lesions, including the neurological outcomes and extent of resection in 136 procedures.

During the resection of brain lesions in critical brain regions that control motor and language functions, surgical neuro-oncology units commonly employ motor and language mapping. This practice aims to preserve the patient’s neurological functioning. To carry out this mapping, specifically trained personnel like neurophysiologists or technicians are essential. They are responsible for the preoperative setup, operation of devices, and crucially, the real-time evaluation of data during surgery [30]. However, it is worth noting that these resources may not always be readily available, especially during night-time procedures or in regions with limited medical infrastructure. Therefore, in our study, we utilized a device that the surgeon can operate independently, which had previously been proven to be effective in emergency situations during the resection of Glioblastoma in two patients facing life-threatening neurological deterioration [31]. Thus, monopolar and bipolar brain mapping can be performed without any further need for external expert staff but by the surgeon alone.

In the present study, we assessed the effectiveness of using the mapping-only device in elective surgical procedures for patients with brain lesions located in critical areas. Our findings indicate that, in 95% of the procedures where we used the mapping-only device without additional monitoring, it was deemed sufficient. Our analysis primarily focused on two key aspects: the success of the lesion resection and the neurological outcomes of the patients.

Monopolar motor mapping was performed in 82% of all procedures and was of particular interest as it could be conducted with relatively straightforward effort. Motor mapping is indicated in order to improve the extent of resection and patients’ outcome [32,33,34]. This approach is commonly employed in surgeries where there is a risk to the motor cortex or corticospinal tract and is often conducted with patients under general anesthesia. Bipolar stimulation is frequently used in patients who are required to undergo language testing during awake surgery, which is a practice commonly described as the standard procedure in the existing literature [16,18,35]. In our cohort, bipolar mapping was also mainly employed for language mapping procedures. For effective language mapping, patients must remain awake during surgery. In our patient group, the majority of procedures were conducted with patients in an awake state (53%). During procedures where bipolar stimulation was applied, a significant proportion of those lesions were found to be located within the left hemisphere, as described in Duffau et al.’s early mapping studies [36,37].

The neurological outcomes in our cohort showed a 2.2% occurrence of permanent neurological deficits, which is a result that is consistent with findings in other, sometimes larger, studies focusing on mapping procedures. In 2008, Sanai et al. described permanent speech deficits in 1.6% of patients in a cohort study of 250 Glioma patients [38]. In our cohort, we observed a tendency toward a higher risk of neurological impairment in patients scheduled for awake surgery but who did not adequately achieve an awake state. This emphasizes the critical importance of thorough awake testing in patients with lesions that potentially affect pathways related to phonological and semantic processing [39].

Motor impairments in our patients occurred only temporarily in a direct postoperative state, and there were no permanent motor deficits; thus we discussed that it is possible to dispense with monitoring procedures in selected cases without posing an additional risk to the patient. In studies where monitoring and mapping techniques were combined, comparable quotas of neurological impairment were seen. Viganò et al. reported 1.9% of permanent deficits using transcranial electric stimulation (TES) motor evoked potential monitoring and direct cortical stimulation [12]. Interestingly, in this study they saw a higher number of false positive results in TES monitoring in patients, depending on tumor localization and patient positioning during surgery. In 2020, Gogos et al. also reported a combined study of “triple motor mapping” with TES, bipolar, and monopolar controls in patients with lesions located near motor pathways. Here, these authors found that, overall, two patients (3.4%) suffered from a new neurological permanent deficit at 6 months, although only one of them showed MEP worsening during resection [28].

As the extent of resection, particularly in high-grade glioma [40,41] but also low-grade glioma [42,43] and even metastasis [44], plays an important role for the overall survival of the patients, a major aim in the treatment of brain tumour patients is to maximize the extent of resection. In cases involving language or motor pathways, this shall be achieved under the preservation of functionability.

In our cohort, gross total resection, defined as residual volume < 0.1 mL (non-measurable) in the postoperative MRI, was achieved in 75 procedures; however, due to mapping results, surgeons were forced to stop the resection in order to preserve the functionability of the patients in 32 cases (25%). In only 20 procedures, an unexpected residual contrast enhancement was seen, with a mean volume of 0.47 mL and a maximum of 2.8 mL in one case. In the literature, there are various limits reported concerning the significant influence of residual tumor volume on overall survival, particularly in glioblastoma patients, ranging from 2 to 5 mL [45,46,47]. The arguably low rate of gross total resection is due to the strictly defined limit of 0.1 mL. If thresholds are applied on our cohorts, which are in line with other reported rates providing advantage for overall survival, we were able to achieve 100% gross total resection, excluding those patients that underwent subtotal resection due to functional limits in the mapping procedures. Therefore, we did not see disadvantages for our patients’ cohort due to waiving additional monitoring.

There are certain limitations in our study that need to be considered. First, it is important to acknowledge that the favorable outcomes observed in our study may be influenced by the exclusion of patients with vascular conflicts. In these cases, MEP and SSEP monitoring is essential for safety reasons. However, it is worth noting that our cohort primarily consisted of patients with highly functional localizations. Second, due to the relatively small sample sizes in some specific subgroups we examined during our data analysis, statistical testing was not feasible and, consequently, was not conducted.

## 5. Conclusions

There is an indisputable increase in safety achieved through the combination of monitoring and mapping techniques. If they are easily and quickly available, these techniques should be aimed for in brain tumour surgeries as described in this paper as a standard procedure. However, we have showed that, deviating from the established combined technical setup of monitoring and mapping possibilities, using a mapping-only device, can achieve comparable results in terms of postoperative deficits and resection outcomes. We have demonstrated that a mapping only technique is safe (2% deficit) and efficient. Brain tumour surgery is performed in many countries around the world. With the help of a mapping-only technique, eloquently located tumors can be treated with great safety and very good resection results. This is a prerequisite for improved outcomes after therapy for aggressive diseases, not least in the case of high-grade infiltrative tumors. Given the simple logistics needed and the affordable price of the technology, we hope that our report promotes the supply and availability of mapping only devices in neurosurgical units under careful consideration of special indications. We think that this could be an absolute gain for brain tumour patients, especially in the field of emergency care, or in health care systems where there might be limited financial—and therefore personnel and technical—access to functional monitoring resources for patients with eloquent brain tumors.

## Figures and Tables

**Table 1:** A brief description of demographic, histopathological, localization and functional tumor involvement data of the cohort.

Age (Year) (n = 131)	
mean	56 [SD ± 16]
range	22–86
Sex (n = 131)	
female	62
male	69
Diagnosis (n = 136)	
Astrocytoma IDH-mutant (2–3)	16
Astrocytoma IDH-mutant (4)	2
Glioblastoma, IDH-Wildtype (4) MGMT –	29
Glioblastoma, IDH-Wildtype (4) MGMT +	28
Oligodendroglioma (2–3)	15
Cerebral metastasis	34
Aggressive NHL	4
Atypical Meningeoma	3
Dysembrioplastic neuroepithelial tumor	1
Ganglioglioma	1
Reactive tissue changes	1
Florid inflammatory demyelinating	1
Localisation (n = 136)	
Left hemisphere	75
Right hemisphere	59
Other	2
Functional Tumour Involvement (n = 136)	
Language only	33
Motor only	59
Combination of language and motor	11
Positive stimulation at tumour margin	31
Not eloquent	2

## Data Availability

Data are available on request by the correspondent author, subject to ethical and legal restrictions.

## Supplementary Materials

The following supporting information can be downloaded at: https://www.mdpi.com/article/10.3390/brainsci13101366/s1, Figure S1: C2 Xtend device and screenshot of intraoperative display of monopolar stimulation.
