# Supplementary figures and images for: Resection of Eloquent Located Brain Tumors by Mapping Only—A Feasibility Study
Source: Brain Sci. 2023 Sep 25;13(10):1366. doi: 10.3390/brainsci13101366 (PMC10605432; doi:10.3390/brainsci13101366)

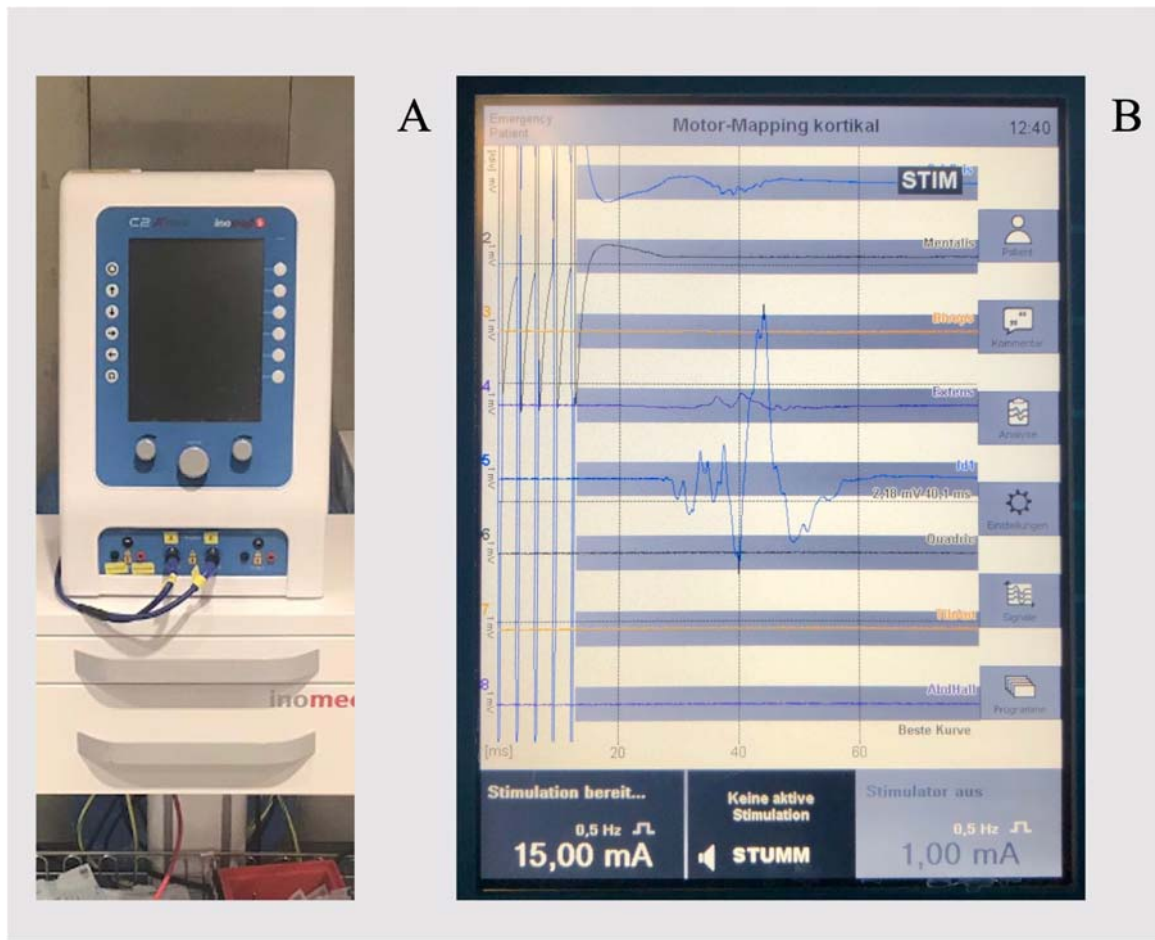

**Figure S1.** C2 Xtend device and screenshot of intraoperative display of monopolar stimulation.

Supplement: Supplementary file 1 [file brainsci-13-01366-s001.zip › brainsci-2596611-supplementary.pdf]
